# Splenic Leukocytes Traffic to the Thyroid and Produce a Novel TSHβ Isoform during Acute *Listeria monocytogenes* Infection in Mice

**DOI:** 10.1371/journal.pone.0146111

**Published:** 2016-01-15

**Authors:** Dina Montufar-Solis, John R. Klein

**Affiliations:** Department of Diagnostic and Biomedical Sciences, School of Dentistry, The University of Texas Health Science Center at Houston, Houston, TX, 77054, United States of America; University of Nebraska Medical center, UNITED STATES

## Abstract

The thyroid stimulating hormone beta-subunit (TSHβ) with TSHα form a glycoprotein hormone that is produced by the anterior pituitary in the hypothalamus-pituitary-thyroid (HPT) axis. Although TSHβ has been known for many years to be made by cells of the immune system, the role of immune system TSH has remained unclear. Recent studies demonstrated that cells of the immune system produce a novel splice variant isoform of TSHβ (TSHβv), but little if any native TSHβ. Here, we show that within three days of systemic infection of mice with *Listeria monocytogenes*, splenic leukocytes synthesized elevated levels of TSHβv. This was accompanied by an influx of CD14^+^, Ly6C^+^, Ly6G^+^ cells into the thyroid of infected mice, and increased levels of intrathyroidal TSHβv gene expression. Adoptive transfer of carboxyfluorescein succinimidyl ester (CFSE)-labeled splenic leukocytes from infected mice into non-infected mice migrated into the thyroid as early as forty-eight hours post-cell transfer, whereas CFSE-labeled cells from non-infected mice failed to traffic to the thyroid. These findings demonstrate for the first time that during bacterial infection peripheral leukocytes produce elevated levels of TSHβv, and that spleen cells traffic to the thyroid where they produce TSHβv intrathyroidally.

## Introduction

Thyroid stimulating hormone (TSH) is a heterodimer consisting of an α-subunit and a β-subunit. Hormone specificity is determined by the β-subunit. There is considerable homology at the gene and protein levels between human and mouse TSHβ [1]. The coding portions of the TSHβ gene are located in mouse exons 4 and 5 and in human exons 2 and 3. TSH, produced by the anterior pituitary, is released into the circulation. Binding of TSH to the TSH receptor (TSHR) in the thyroid triggers secretion of thyroxine (T4), which is converted by deiodinase into triiodothyronine (T3), the more biologically active form of thyroid hormone. Thyroid hormones are essential for maintaining basal metabolism, growth, development, mood, and cognition.

Although it has been known for over three decades that TSH is produced by cells of the immune system [2, 3], the functional significance of that has remained elusive. Studies in our laboratory demonstrated that whereas the full-length native form of TSHβ (coded from mouse exons 4 and 5) is produced by the anterior pituitary, cells of the mouse immune system make a novel splice variant form of TSHβ (TSHβv) using the 3’ portion of intron 4 and exon 5, with the exclusion of exon 4 [4] TSHβv is expressed in bone marrow (BM) cells and the thyroid of mice [4] and humans [5]. Studies from other laboratories have confirmed TSHβv synthesis in macrophages [6]. TSHβv dimerizes with TSHα [7], and is a secreted protein that delivers an intracellular cAMP signal upon docking to the TSHR [4, 6]. Recent studies have linked the TSHβv to Hashimoto’s thyroiditis [7].

Little is currently known about the role of immune system-derived TSHβv during infection. To that end, we used a conventional model of systemic *L*. *monocytogenes* infection in mice. Here, we demonstrate: (i) that TSHβv expression levels are increased in splenic leukocytes following *L*. *monocytogenes* infection, (ii) that CD14^+^, Ly6C^+^, Ly6G^+^ cells infiltrate the thyroid of infected mice, (iii) that TSHβv is produced at elevated levels in the thyroid during infection, and (iv) that splenic leukocytes from infected mice migrate to the thyroid of non-infected mice, thus demonstrating an active trafficking process of TSHβv-producing cells into the thyroid during infection. These findings provide new insights into the involvement of TSHβv during periods of immune stress due to bacterial infection.

## Materials and Methods

### Mice, bacteria, and infection

Adult female C57BL/6 mice, 6–8 weeks of age, were purchased from Harlan (Indianapolis, IN). Animals were used in accordance with the University of Texas Health Science Center at Houston Institutional Animal Welfare Committee permit No. HSC-AWC-12-039, which specifically pertains to this study. *L*. *monocytogenes* serotype 4b was obtained from American Type Culture Collection, Manassas, VA. Bacteria were grown in brain heart infusion broth and titered by serial dilution on brain heart infusion agar plates (Fisher Scientific, Pittsburgh, PA). Animals were infected by i.p. injection of 1.79x10^9^ CFU *L*. *monocytogenes*. That dose was selected because C57BL/6 mice are more resistant to infection than most other strains [8], because doses for i.p. infection range from 10^6^−10^9^ CFU as reported by others [9, 10], and because of a short duration of infection of 3 days was used. To alleviate pain from virus infection or adoptive cell transfer (below), mice were treated with Buprenorphine, 0.05–0.1 mg/kg body weight given i.p. 2–3 times per day as needed.

### Quantitative real-time PCR (qRT-PCR)

qRT-PCR was done as previously reported by our laboratory [11, 12] using a StepOnePlus real-time thermal cycler and software (Life Technologies, Applied Biosystems, Grand Island, NY). Relative gene expression was normalized to GAPDH. Primers were:

TSHβ native forward: 5’- AAGAGCTCGGGTTGTTCAAA -3’

TSHβ native reverse: 5’- AACCAGATTGCACTGCTATTGAA-3’

TSHβv forward: 5’- ATCATGTTAAGATCTCTTTTCTTT-3’

TSHβv reverse: 5’- AACCAGATTGCACTGCTATTGAA-3’

GAPDH forward: 5’-GTGTTCCTACCCCCAATGTGT-3’

GAPDH reverse: 5’-ATTGTCATACCAGGAAATGAGCTT-3’

To visualize PCR products, samples from qRT-PCR reactions were run in a 2% agarose gel as reported [4].

### Antibodies, flow cytometry, and immunofluorescence microscopy

Goat M-16 anti-TSHβ antibody (Santa Cruz Biotechnology, sc-7815, Dallas, TX) to the C-terminus region of the mouse TSHβ polypeptide coded for by exon 5, the immunoreactive portion of TSHβv, was used for intracellular staining of splenic leukocytes and in the enzyme-linked assay (EIA). Monoclonal anti-mouse TSHβ (1B11) antibody made in our laboratory to amino acids 113–124 in the C-terminus region of mouse TSHβ coded for by exon 5 [13], and a polyvalent rabbit antibody made for our laboratory (Thermo Scientific, Pierce Biotechnology, Rockford, IL) to amino acids 25–35 in the N-terminus region of mouse TSHβ polypeptide coded for by exon 4 was used for intracellular staining of AM cells. Antibodies 25–35 and 113–124 were biotinylated in our laboratory using a ProtOn Biotin Labeling Kit (Vector Laboratories, Burlingame, CA).

One-color surface staining of BM cells was done using FITC-anti-CD45-leukocyte common antigen (CD45-LCA) antibody (RA3-6B2, eBioscience, San Diego, CA), or FITC-IgG control antibody (eBM2a, eBioscience). For intracellular staining, membranes were permeabilized for 20 min at 4°C with cytofix/cytoperm (BD Bioscience, San Diego, CA), washed x2 with permwash (BD Bioscience), reacted with M-16 anti-TSHβ antibody or normal goat IgG control antibody (Santa Cruz, sc-2028) at 4°C for 30 min, washed x1 with permwash, and reacted at 4°C for 15 min with FITC-donkey anti-goat antibody (Santa Cruz, sc-2024).

Two-color staining of splenic leukocytes was done using: PE-anti-CD14 (Sa2-8), PE-anti-Ly6C (HK1.4), and PE-anti-F4/80 (BM8) (eBioscience, all reagents), PE-anti-CD11b (M1/70) and PE-anti-CD11c (HL3) (BD Bioscience, all reagents), PE-anti-Ly6G (1A8) (BioLegend, San Diego, CA), and PE IgG control antibody (eBRG1, eBioscience). Intracellular staining for TSHβ was done using M-16 anti-TSHβ antibody, and normal goat IgG control antibody (Santa Cruz, sc-2028), followed by FITC-donkey anti-goat control antibody (Santa Cruz, sc-2024) using the staining protocol described above.

AM cells were provided by Dr. Chinnaswamy Jagannath, Department of Pathology, the University of Texas Health Science Center at Houston. One-color surface staining was done using PE-anti-CD14 antibody, PE-anti-TLR2 antibody, or PE IgG control antibody. One-color intracellular staining of permeabilized membranes, described above, was done for TSHβv by reacting cells with biotinylated antibody 25–35, biotinylated antibody 113–124, or biotinylated IgG control antibody (cat. No. 553273, BD Bioscience) followed by streptavidin-APC (eBioscience). Cell fluorescence was determined on a FACSCalibur flow cytometer with CellQuest software (BD Bioscience).

For tissue immunofluorescence microscopy, thyroids were snap frozen in liquid nitrogen, mounted in Tissue-Tek® OCT Compound, and 4μM sections were cut on a Research Cryostat (Leica CM3050S, Buffalo Grove, IL). Sections were fixed in acetone, hydrated in PBS, blocked with 10% fetal bovine serum in PBS, incubated with FITC-labeled antibodies, washed in PBS, and mounted onto slides using Fluoro Gel with DABCO (Electron Microscopy Diatome No. 17985–01). DAPI (Life Technologies) was added for a final concentration of 1:1000. Antibodies used were FITC-IgG control antibody (eB149/10H5), FITC-anti-CD11b (M1/70), FITC-anti-CD11c (N418), FITC-anti-CD14 (Sa2-8), and FITC-anti-F4/80 (BM8) (eBioscience, all reagents), FITC-anti-Ly-6C (HK1.4) and FITC anti-Ly-6G (1A8) (BioLegend, all reagents). Tissue sections were examined on a Nikon Eclipse E400 microscope for fluorescence imaging using NIS Elements BR- 3.2 software.

### Cell culture and EIA

1x10^6^ AM/ml were cultured in 24 well cell culture plates (Genesee Scientific, San Diego, CA) at 37°C in 5% CO_2_ in RPMI-1640 media with 10% FBS, penicillin/streptomycin, and 2μM L-glutamine. For determination of secreted TSHβ, AM cells were cultured with 100, 50, 25, 12.5 μg/ml zymosan (ICN Pharmaceuticals, Aurora, OH), 100 ng/ml *E*. *coli* LPS (Sigma-Aldrich Chemicals, St. Louis, MO) [14], or with soluble anti-CD14 or anti-TLR2 antibody. Following 1 week in vitro culture with zymosan, cells were fixed in ice cold 90% MEOH for 30 min, washed x3 with PBS, reacted with 1:100 M-16 anti-TSHβ antibody at 4°C for 30 min, washed x3 with PBS, and reacted with donkey anti-goat Texas Red (Santa Cruz, sc-2783) at 4°C for 30 min, washed x3 with PBS, and reacted with DAPI. Cells were examined using a Nikon Eclipse T1 inverted microscope.

A mouse TSHβ EIA was used in which dilutions of culture supernatants, or dilutions of recombinant TSHβ [4], were coated overnight at 4°C onto Costar EIA/RIA stripwell high-binding polystyrene plates (Corning, Corning, NY), washed x3 in PBS with 0.05% tween with a Multi-Wash Advantage Microtiter Plate Washer (TriContinent Scientific, Grass Valley, CA), blocked for 1 hr at room temperature with 2% BSA/PBS, washed x3, incubated at room temperature for 1 hr with 1:100 of M-16 anti-TSHβ antibody, washed x3, incubated at room temperature for 30 min with HRP-donkey anti-goat antibody (Santa Cruz, sc-2020), washed x4, reacted for 15 min with TMB substrate (eBioscience). The reaction was stopped with 1 M phosphoric acid; the optical density was determined using a Vmax Kinetic Microplate Reader (Molecular Devices, Sunnyvale, CA). The concentration of TSHβ was predicted from a standard curve generated using recombinant mouse TSHβ [4].

### CFSE labeling and adoptive cell transfer

Spleen cells were recovered from euthanized C57BL/6 mice 3 days post-*L*. *monocytogenes* infection or injection with PBS. Erythrocytes were lysed and cells were stained with 10 μM CFSE (Invitrogen; Carlsbad, CA, USA) prepared in DMSO. Cells were incubated for 15 min at 37°C in 5% CO_2_ environment, washed in PBS, and pelleted. 9x10^6^ cells suspended in 100 μl physiological saline and injected retro-orbitally [15] into isoflurane-anesthetized non-infected normal C57BL/6 mice. Eyes were treated with a neomycin and polymyxin B sulfates, bacitracin zinc, and hydrocortisone ophthalmic ointment USP. 24 and 48 hr post-transfer, animals were euthanized, the thyroid was recovered, sectioned, and examined by immunofluorescence microscopy for CFSE^+^ cells.

### Statistical analyses

A two-tailed Student’s t-test was used for determination of statistical significance.

## Results

### Bone marrow cells in normal and *L*. *monocytogenes*-infected mice produce TSHβv

**Fig 1:**
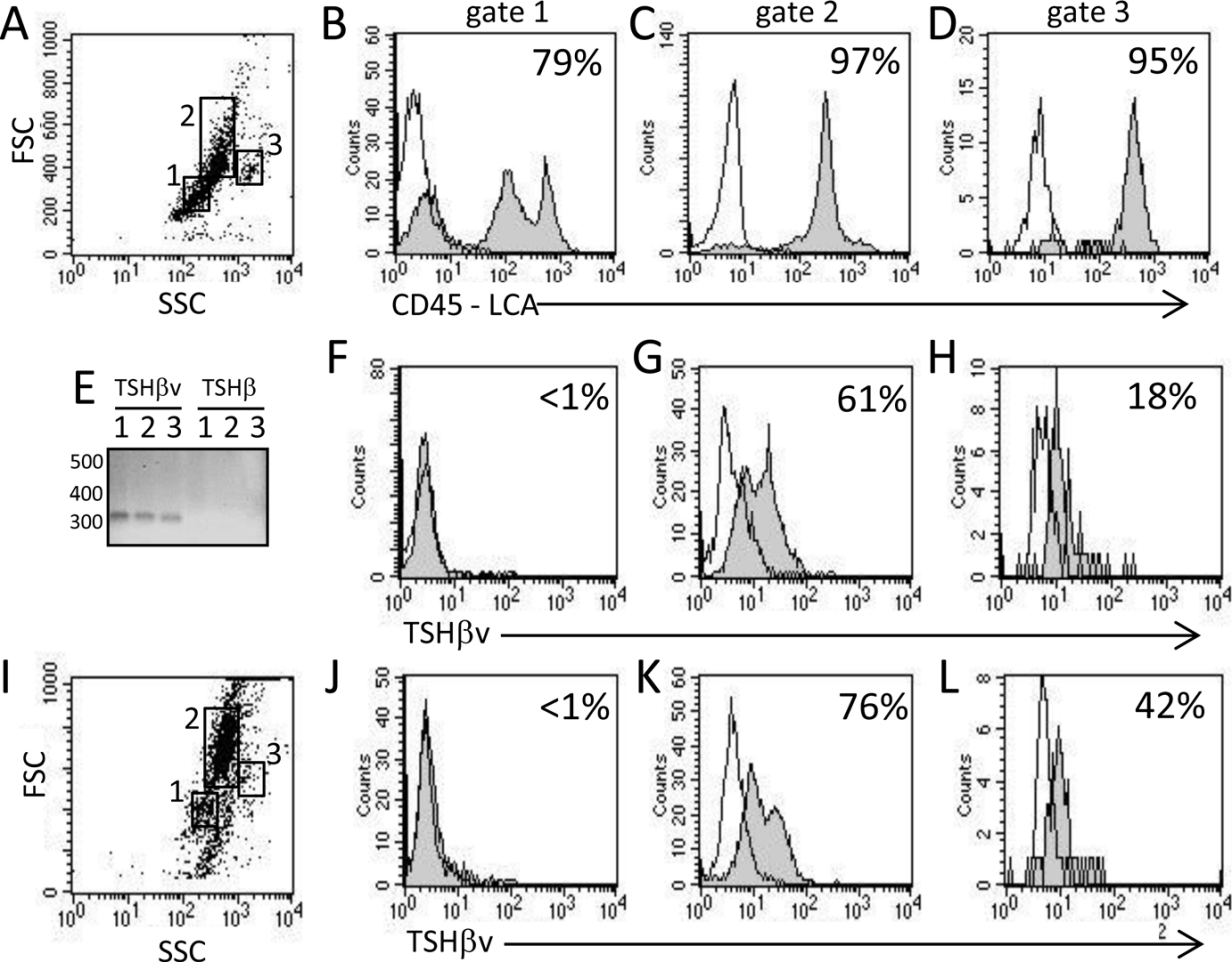
The TSHβv is produced by BM cells in normal and *L*. *monocytogenes-*infected mice. (**A**) Characterization of BM cells from normal non-infected mice according to FSC (cell size) and SSC (granularity). Percentage of CD45-LCA^+^ cells in (**B**) gate 1, (**C**) gate 2, and (**D**) gate 3. (**E**) qRT-PCR analysis of normal BM cells for TSHβv and native TSHβ gene expression. 1, 2, 3 are BM cells from each of 3 mice. Intracellular TSHβ M-16 staining of BM cells from normal non-infected mice in (**F**) gate 1, (**G**) gate 2, and (**H**) gate 3 in histogram A. (**I**) Characterization of BM cells from mice 3 days post-i.p. infection according to FSC and SSC. Intracellular TSHβ M-16 staining of BM cells from infected mice in (**J**) gate 1, (**K**) gate 2, and (**L**) gate 3 in histogram in I. Histogram data are representative of three independent experiments. Background staining (unfilled) in overlaid histograms is the reactivity of IgG control antibody.

BM cells from normal mice were defined according to forward scatter (FSC) and side scatter (SSC) and by expression of the CD45 leukocyte common antigen (LCA). This consisted of CD45^+^ typical of lymphocyte precursors (Fig 1A gate 1, Fig 1B), CD45^+^ typical of monocyte/macrophage precursors (Fig 1A gate 2, Fig 1C), and CD45^+^ typical of granulocyte precursors (Fig 1A gate 3, Fig 1D). We previously reported gene expression of TSHβv but not native TSHβ in BM cells [4]. Similar findings were obtained in the present study as seen by gene expression of TSHβv but not native TSHβ in normal BM cells (Fig 1E). Those findings are important because they confirm that intracellular staining of BM cells using antibody M-16, which is reactive with the C terminus region of the TSHβ polypeptide coded for by exon 5, but does not react with the TSHβ polypeptide region coded for by exon 4, reflects the selective expression of TSHβv in BM cells.

Staining of BM cells from normal non-infected mice revealed that TSHβv is not associated with the lymphocyte precursor population (Fig 1F), that is heavily associated with the monocyte/macrophage precursor population (Fig 1G), and that it is minimally associated with the granulocyte precursor population (Fig 1H). Mice were infected i.p. with *L*. *monocytogenes* as described in the Materials and Methods. Three days after infection, TSHβv expression in lymphocyte precursors remained negative (Fig 1J), although there was a modest increases in TSHβv^+^ in monocyte precursors (Fig 1K) and granulocyte precursors (Fig 1L) compared to non-infected mice. Those findings identify differential expression patterns of TSHβv in BM cells, which is principally linked to the monocyte/macrophage precursor population.

### TSHβv expression increases in splenic leukocytes during *L*. *monocytogenes* infection

**Fig 2:**
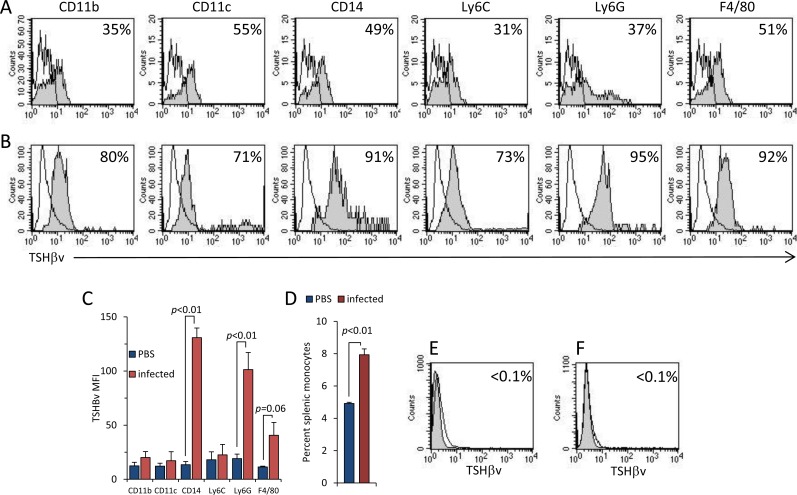
*L*. *monocytogenes* infection results in increased TSHβv production in splenic leukocytes. Immunoreactivity of M-16 for TSHβv expression in CD11b^+^, CD11c^+^, CD14^+^, Ly6C^+^, Ly6G^+^, and F4/80^+^ spleen cells from (**A**) normal non-infected mice and (**B**) *L*. *monocytogenes*–infected mice. (**C**) MFI of TSHβv of intracellular staining of cells from PBS-injected or *L*. *monocytogenes*-infected mice. (**D**) Percent splenic monocytes (gates 2 in Fig 1A and 1I) from PBS-injected and *L*. *monocytogenes*-infected mice. TSHβv intracellular staining of splenic lymphocytes from (**E**) normal PBS-infected and (**F**) *L*. *monocytogenes*-infected mice. 2–3 mice were used per experimental set. Infected mice were used 3 days post-infection. Background staining (unfilled) in overlaid histograms is the reactivity of IgG control antibody.

To assess the changes that occur in the expression of TSHβv in peripheral leukocytes of *L*. *monocytogenes*-infected mice, spleen cells were stained for six markers commonly expressed on myeloid cells (CD11b, CD11c, CD14, Ly6C, Ly6G, and F4/80). Two-color staining was done as described in the Materials and Methods in conjunction with anti-TSHβ M-16 antibody. Spleen cells were analyzed 3 days post-infection by flow cytometry in cells in gates 2 (shown in Fig 1). There was an increase in TSHβv expression in all six phenotypic populations of infected mice (Fig 2 panel B) compared to PBS-injected control mice (Fig 2 panel A). This was particularly evident for CD14^+,^ Ly6G^+^, and F4/80^+^ cells as seen by higher mean fluorescence intensity (MFI) (Fig 2C). Additionally, there was an overall increase in the total percent of splenic mononuclear cells in infected mice (Fig 2D). TSHβv expression was not detected in splenic lymphocytes in either PBS-injected (Fig 2E) or *L*. *monocytogenes*-infected animals (Fig 2F).

**Fig 3:**
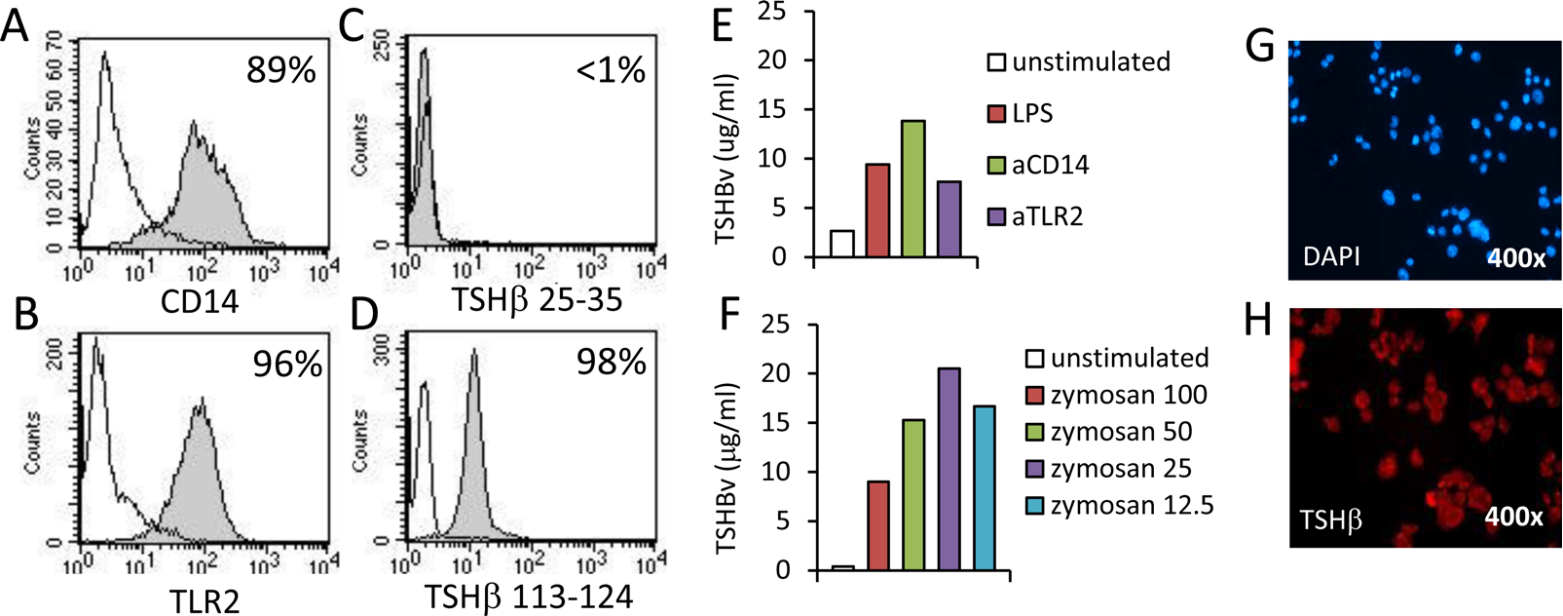
Multiple stiumlatory signals induce TSHβv synthesis in mouse monocyte/macrophage cells. Expression of (**A**) CD14 and (**B**) TLR2 on mouse AM cells. Staining of AM cells with (**C**) antibody to TSHβ peptide 25–35 in the N-terminus region of mouse TSHβ, and (**D**) antibody to TSHβv peptide 113–124 in the C-terminus region of mouse TSHβ. (**E**) Quantification of TSHβ in cell culture supernatants of AM cells cultured for 24 hr with LPS, anti-CD14 antibody, or anti-TLR2 antibody, or with (**F**) graded doses of zymosan as determined by EIA. (**G**) DAPI and (**H**) M-16 TSHβ staining of AM cells 1 week post-culture with zymosan as determined by immunofluorescence microscopy. 2–3 mice were used per experimental set. Infected mice were used 3 days post-infection. Background staining (unfilled) in overlaid histograms is the reactivity of IgG control antibody.

The mouse AM macrophage cell line was used to study inductive requirements of TSHβv synthesis. AM cells are CD14^+^ (Fig 3A) and TLR2^+^ (Fig 3B) and did not produce native TSHβ (Fig 3C) but did produce TSHβv (Fig 3D). When stimulated with LPS, or with anti-CD14 or anti-TLR2 antibodies, TSHβv secretion increased in AM cells compared to non-stimulated cells as determined by EIA (Fig 3E). Induction of phagocytosis in AM cells by zymosan resulted in increased secretion of TSHβv in a dose dependent manner (Fig 3F). Zymosan-stimulated AM cells were TSHβv^+^ for one week post-stimulation (Fig 1G and 1H). Those findings collectively indicate that the peripheral immune response of splenic leukocytes to *L*. *monocytogenes* infection is accompanied by increased TSHβv synthesis, and that multiple immune stimuli can induce TSHβv synthesis in mouse monocytes.

### Splenic leukocytes in *L*. *monocytogenes*-infected mice traffic to the thyroid and produce TSHβv intrathyroidally

**Fig 4:**
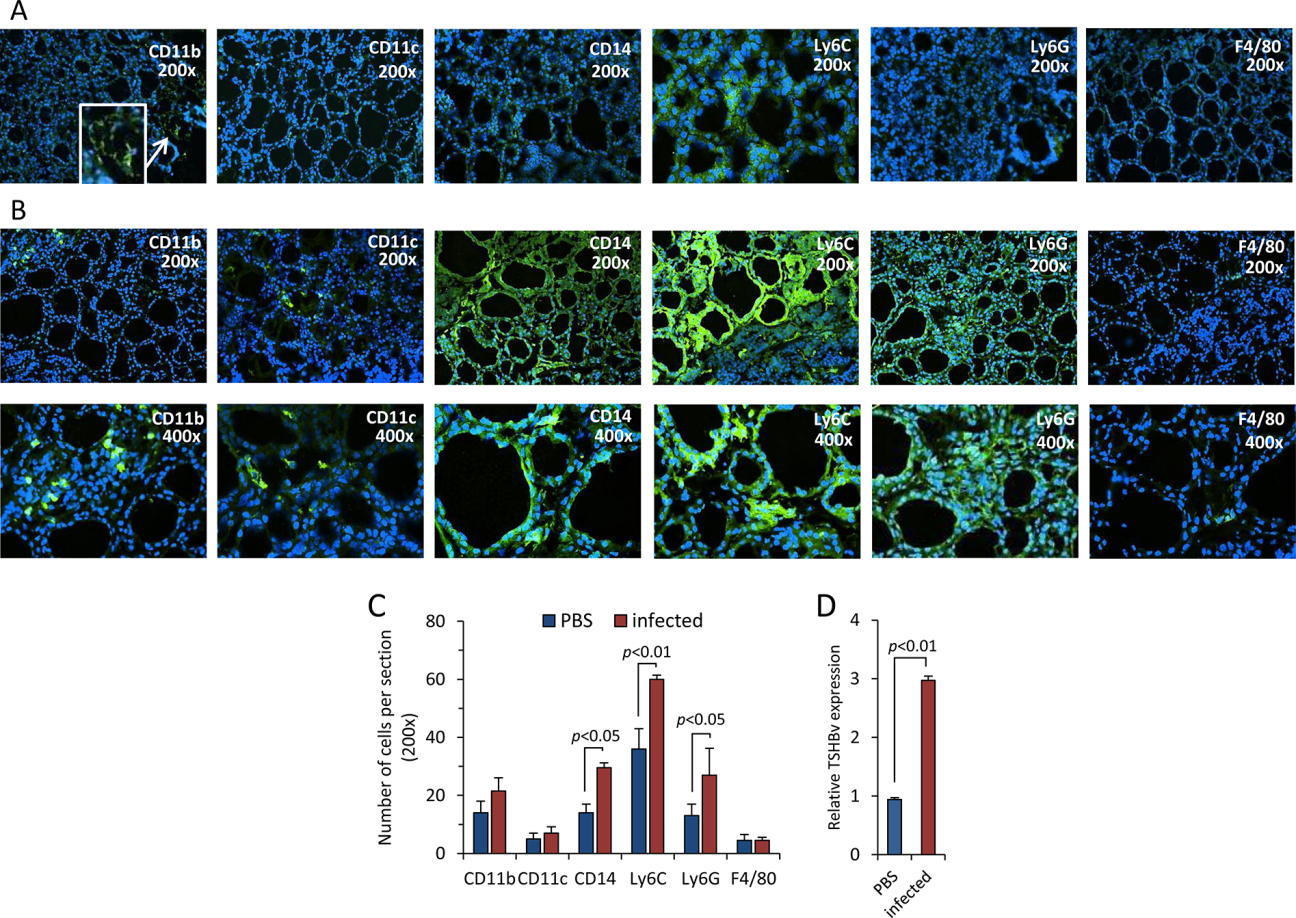
Influx of CD14^+^, Ly6C^+^, Ly6G^+^ cells into the thyroid of mice following *L*. *monocytogenes* infection with elevated TSHβv expression in the thyroid. Thyroid tissue frozen sections from (**A**) PBS-injected mice were and (**B**) *L*. *monocytogenes*-infected mice stained with antibody to CD11b, CD11c, CD14, Ly6C, Ly6G, and F4/80 3 days post-infection as described in the Materials and Methods. (**C**) Numbers of respective cells counted from 3 independent stained sections of PBS-injected and *L*. *monocytogenes*-infected mice 3 days post-infection. (**D**) TSHβv gene expression in the thyroid of PBS-injected and *L*. *monocytogenes*–infected mice 3 days post-infection.

Mice were infected with *L*. *monocytogenes* or injected with PBS. Three days later, thyroids were recovered, snap frozen, stained for expression of CD11b, CD11c, CD14, Ly6C, Ly6G, and F4/80, and examined by fluorescence microscopy. Compared to thyroid tissues from PBS-injected mice (Fig 4 panel A, and Fig 4C), there was a significant increase in the influx of CD14^+^, Ly6C^+^, and Ly6G^+^ cells into the thyroid of infected mice (Fig 4 panel B, and Fig 4C). This was accompanied by an increase in TSHβv gene expression in the thyroid (Fig 4D).

**Fig 5:**
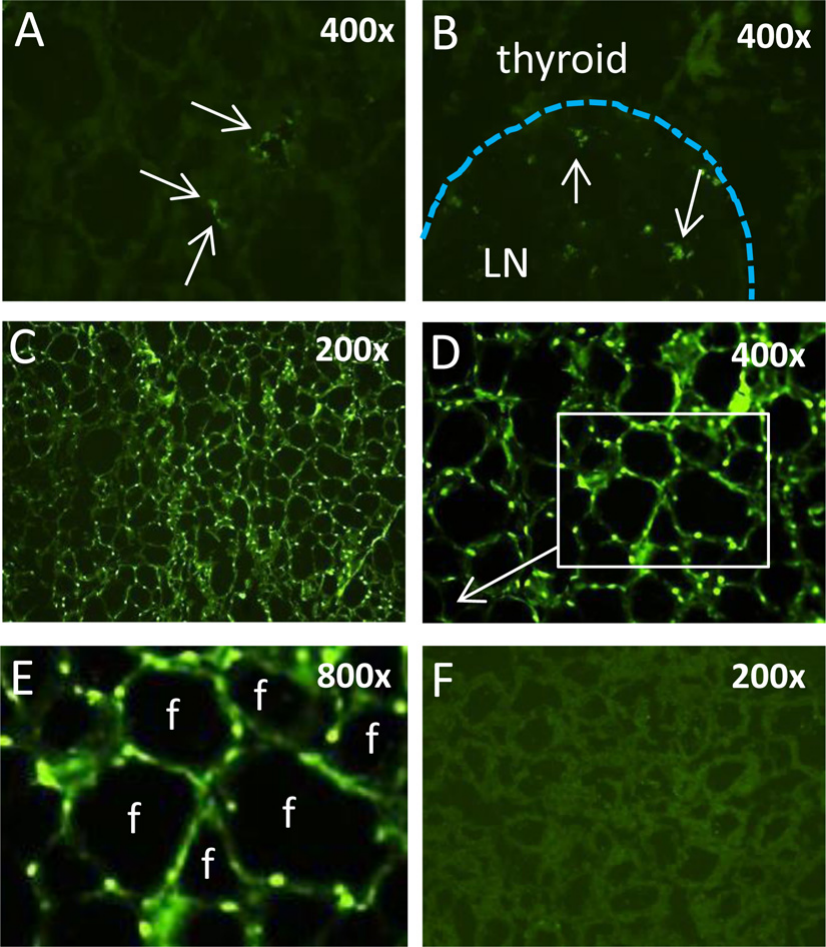
Splenic leukocytes from *L*. *monocytogenes*-infected mice, but not normal mice, traffic to the thyroid. Immunofluorescence analysis of (**A**) the thyroid and (**B**) a thyroid perivascular lymph node from a non-infected mouse 24 hr post-cell transfer of CFSE-labeled splenic leukocytes from a *L*. *monocytogenes*-infected mouse. Thyroid of a non-infected mouse 48 hr post-transfer of CFSE-labeled spleen cells from a *L*. *monocytogenes*-infected mouse (**C**) 200x, (**D**) 400x, (**E**) 800x, boxed area in D. (**F**) Thyroid of a non-infected mouse injected with CFSE-labeled spleen cells from a non-infected mouse.

To determine if splenic leukocytes from *L*. *monocytogenes*-infected mice actively traffic to the thyroid, spleen cells were collected from mice infected for three days with *L*. *monocytogenes*. Erythrocytes were lysed, cells were labeled with carboxyfluorescein succinimidyl ester (CFSE), and 9x10^6^ cells were transferred by retro-orbital injection [15] into normal non-infected mice. Recipient animals were euthanized 24 and 48 hr post-cell transfer, the thyroid was removed and examined by fluorescence microscopy for evidence of cell trafficking based on the presence of intrathyroidal CFSE^+^ cells. 24 hr post-transfer, CFSE^+^ cells were sparsely dispersed throughout the thyroid (Fig 5A) and pericapsular lymph nodes (Fig 5B) of recipient mice. Notably, the thyroid of mice that had been injected with spleen cells from *L*. *monocytogenes*-infected mice animals heavily infiltrated with CFSE^+^ cells 48 hr post-cell transfer; nearly all thyroid follicles were surrounded by at least one and in many cases 3–4, CFSE^+^ cells (Fig 5C–5E). In contrast, CFSE-labeled cells from normal non-infected donor mice did not traffic to the thyroid (Fig 5F). These findings provide strong evidence of an active trafficking process of peripheral leukocytes into the thyroid during acute *L*. *monocytogenes* infection.

## Discussion

Despite longstanding evidence linking TSH production to cells of the immune system, the significance of that has remained elusive. Yet, it is interesting that both the endocrine system and the immune system share a number of common features. Both are broadly dispersed throughout the body, consisting of receptors and ligands used in positive and negative regulation. A hallmark of both immune and endocrine responses is communication with the external environment, such as situations that detect and respond to metabolic stress in the case of the endocrine system, and foreign antigen exposure in the case of the immune system. It is thus not surprising that reciprocal immune-endocrine communication networks exist that serve to enhance the functionality of each other. The identification of a TSHβ splice variant adds a level of complexity and intrigue to those activities, in particular because of the findings that TSHβv is the exclusive form of TSHβ made by the immune system, and because of its potential link to thyroid disease such as Hashimoto’s thyroiditis [4, 6, 7, 16].

In extending those studies, we sought to determine the extent to which the responses of immune system-derived TSHβv are altered during acute exposure to pathogenic bacteria. Here, we formally demonstrate that challenge with *L*. *monocytogenes* induces increased levels of TSHβv synthesis across a range of splenic leukocyte subsets, although the cells that trafficked to the thyroid during infection favored CD14^+^, Ly6C^+^, Ly6G^+^ cells. These may represent discrete subsets bearing those markers, or may be select groups, some of which have yet to be defined phenotypically. One possible distinction pertains to the classification of peripheral monocytes as classical vs. non-classical cells based on the expression of CD14 and selected chemokines and their receptors [17]. Interestingly, CD14^+^ non-classical monocytes can be activated by TLR2 stimulation [18], a finding consistent with the observation of TLR2-induced TSHβv synthesis from AM cells as described here. Additionally, non-classical monocytes traffic to non-inflamed peripheral sites such as the spleen, liver, and lungs [19, 20], and thus may be candidates for the cells that homed to the thyroid in the present study. Although it also has been shown that non-classical monocytes increase more slowly in the circulation during chronic infection [19, 21, 22], the reverse occurred for *L*. *monocytogenes* infection [20]. These findings collectively point to a dynamic process of cell trafficking into and through non-lymphoid tissues.

Studies from other laboratories have linked TSHβv synthesis to F4/80^+^ macrophages, particularly M2 macrophages generated from BM cells [6]. Although TSHβv was also produced by splenic F4/80^+^ cells in our study (Fig 2, panel B), few F4/80^+^ cells were present in the thyroid of either normal or infected mice. It has been reported that F4/80 antibodies may be unreliable for flow cytometric identification of macrophages, and that combinations of Ly6C and Ly6G are more suitable indicators of those cells [23], a property that would be consistent with the increase in Ly6C^+^ Ly6G^+^ cells in the thyroid as reported here.

That infection induced an active process of intrathyroidal cell trafficking was confirmed by adoptive transfer of spleen cells from infected mice into non-infected animals in which there was a marked influx of cells into the thyroid 48 hr post-transfer. The fact that spleen cells from non-infected animals did not traffic to the thyroid indicates that a regulated mechanism exists for directing TSHβv-producing cells into the thyroid. Moreover, given that in these experiments trafficking occurred by spleen cells from infected mice independent of the infection status of recipient animals, the signal necessary to drive the response must be at the level of the TSHβv-producing cells, possibly due to the upregulation of a receptor for a ligand expressed in the thyroid. Potential migration-inducing markers on spleen cells include chemokines and chemokine ligands and receptors such as CCR2 on spleen cells and CCL2 on thyrocytes [24]. Other possible monocyte molecules that may facilitate migration would be CX_3_CL1 [25] and CXCL12 [26], both of which may facilitate transendothelial migration of cells. Studies are currently underway to explore those and other possible mechanisms associated with TSHβv cell migratory responses.

Although the functional role of TSHβv synthesis in the thyroid remains to be determined, we have proposed that the contribution of immune system TSHβv may be to micro-regulate thyroid hormone output during times of immune stress [27–30]. Accordingly, local intrathyroidal production of TSHβv by splenic leukocytes would compete with native TSHβ for binding to the TSHR, resulting in a weakened signal for thyroid hormone synthesis. The net effect of this would be to curtail host metabolic responses, leading to energy conservation during the recovery phase of infection. The findings reported lend credence to this scenario during bacterial infection.
